# Supplementary material for: Are carbon emissions trading and green financial instruments synergistic? -Comprehensive quantitative research based on content analysis
Source: PLoS One. 2024 Mar 7;19(3):e0298601. doi: 10.1371/journal.pone.0298601 (PMC10919601; doi:10.1371/journal.pone.0298601)
Supplement: S1 Data — (ZIP) [file pone.0298601.s001.zip › Data/地级市Z_F工作报告.zip!/░▓╗╒/╗┤▒▒╩╨/└┤╫╘╣1⁄2╓┌║┼í╛┬φ┐╦╩2╛▌═°í┐_2018.docx]

各位代表：

现在，我代表市人民政府，向大会报告政府工作，请予审议，并请市政协委员和其他列席人员提出意见。

****一、攻坚克难、顽强拼搏的奋进历程****

2017年是淮北市经济社会发展进程中极不平凡的一年。一年来，在省委、省政府和市委的坚强领导下，市政府团结带领全市人民，深入学习贯彻习近平新时代中国特色社会主义思想，围绕中国碳谷·绿金淮北战略和“一二三四五”总体发展思路，统筹推进抓重点，全力以赴抓落实。预计，全年生产总值增长7.6%，固定资产投资增长10.6%，社会消费品零售总额增长11.3%，城乡居民人均可支配收入分别增长7.5%、8%，城镇登记失业率2.59%。财政收入继2012年后再次突破百亿元大关，达到107.6亿元，增长15.3%，创历史最好水平，圆满完成市十五届人大七次会议确定的各项目标任务。

经过全市上下的共同努力，一批事关淮北长远发展的重大项目实现新突破，一批惠及全市人民福祉的民生建设迈上新台阶，一批历史遗留问题破解取得新成效，一批绩效考核重点工作和经济指标进入全省第一方阵。

****（一）深入推进“去降补”，供给侧结构性改革成效明显。****岱河煤矿、刘桥一矿、金石矿业顺利关闭，退出产能305万吨，妥善安置职工9959人，“三供一业”分离移交有序推进。全市商品房待售面积同比下降8.4%。开展降成本减轻实体经济企业负担专项行动，全市结构性减税9亿元。开展电力直接交易和天然气大用户直供，为企业节约成本1.2亿元。强化项目带动，加大补短板力度，全年实施各类重点项目537个、完成投资465亿元。组织开展“四送一服”“三个一百”帮联企业专项行动，累计为企业解决实际问题299个，为267家中小企业提供过桥贷款，新型政银担业务在保余额21.7亿元，“新三板”、四板市场挂牌企业总数突破100家。淮北矿业股份公司重组上市取得重大突破。全省首单市场化债转股资金落地淮北，累计投放债转股资金76.2亿元，企业杠杆率有效降低。淮北矿业、皖北煤电、淮海实业在渡危求进中革新图强，为我市经济持续向好作出了重要贡献。

****（二）大力实施创新驱动战略，转型升级步伐不断加快。****出台促进经济平稳健康发展“30条”、推动“三重一创”建设“10条”等系列政策，濉溪铝基高端金属材料产业基地通过省评估考核，相邦陶铝新材料、科宝生物医药中间体获批省重大新兴产业专项，上海交大安徽陶铝新材料研究院正式成立。战略性新兴产业产值增长20.1%。新认定国家高新技术企业22家，高新技术产业增加值增长15.3%。东华（淮北）全国中小企业云服务智慧产业园签约落地。扎实推进大众创业、万众创新，累计建成省级孵化器1家、众创空间4家，创新创业服务平台更加完善。

“十大工业项目”进展顺利，其中，华润金蟾中药配方颗粒制剂、金龙AG玻璃、花园新材料一期建成投产，平山电厂二期、口子产业园、完美生物、华孚产业园开工建设。实施海螺水泥、长治电子等技改项目141个，理士电源、曦强乳业等77个项目获制造强省政策支持。全市新增规模以上工业企业79家，规模以上工业增加值增长8.8%，规模以上工业企业利税、利润增幅全省领先。粮食生产实现“十四连丰”，全年总产129.5万吨，小麦平均单产创历史新高。培育市级以上农业产业化龙头企业141家，农产品加工产值占农业总产值的比重居全省首位。传化智联城市物流中心、智汇电商产业园正式运营，中农批淮北大市场、青龙山现代物流基地加快建设，获批省级服务业集聚区4家。濉溪县电商产业园获评省级示范园区。南湖景区跻身国家4A级旅游景区，旅游业加快发展的载体进一步夯实。

****（三）持续深化改革开放，活力动力竞相迸发。****“放管服”改革取得显著成效，在全国首创“互联网+实名制”监管模式、首个开展“机构编制二维码”试点建设及应用。发出全省首张“多证合一”营业执照，五位一体“双随机一公开”监管新模式获省政府通报表扬。全年新登记私营企业4964户、个体工商户16309户，分别增长20.7%、28.3%。不动产登记全面提速，日均受理量由140件提高到288件，企业和群众满意度不断提升。统计管理体制改革不断深化，服务发展能力显著增强。8个农村集体产权制度改革试点稳步推进，全市累计完成土地确权登记颁证面积233万亩。金西域生态休闲农业项目入选全国首批农业PPP试点。市金控集团正式成立，主营业务收入突破50亿元。我市荣获全国城市信用建设创新奖。

出台招商“10条”，大力开展产业招商、精准招商、以商招商。全年新签约规模项目285个，新开工规模项目202个，其中5亿元以上项目25个，华孚绿尚小镇、天悦电子等重大项目开工建设。新型煤化工基地十大项目集中开工，产业链招商实现新突破。市开发区与河北荣盛集团签订整体开发建设经营协议，通过PPP模式建设碳谷产业新城。大力发展非公经济，民营经济增加值占生产总值比重达58%。积极发展对外贸易，全市完成进出口总额6.2亿美元。举办2017淮北食品工业博览会、石榴文化旅游节、段园葡萄采摘节等活动，城市知名度进一步提升。淮萧客车联络线建成运营，我市正式进入高铁时代，融入京沪“四小时经济圈”，全市人民出行更加便捷，对外开放迎来重大机遇。

****（四）加大城乡建设投入，城市形象大幅提升。****“十大城建工程”全面推进，其中，棚户区改造、东湖景区、京台高速出入口景观提升工程全面完成，市规划展示馆和档案馆开工建设，骨干路网、中湖景区、黑臭水体治理加快推进。完善城乡规划体系，编制完成《淮北市空间规划》《东部新城城市设计》，稳步推进《淮北市城市总体规划（2016-2035年）》修编工作。我市被列为国家第三批城市“双修”试点市、省级生态网络规划试点市。

全年完成城乡基础设施投资161亿元，同比增长53.5%。新建交通干线、城市道路111.7公里，煤化工快速通道、梧桐中路、创新大道、龙山南路等一批主干道路建成通车，S101合相路二期、S254古毛路二期、S235濉唐路二期开工建设，东部新城、高铁新区路网加快推进，符夹铁路立交桥基本建成。梅苑中路、桂苑中路等断头路顺利打通，迎宾路、梧桐路等交通路口渠化改造完成，道路拥堵逐步缓解。推进老城区雨污分流，完善污水主干管网12公里。新增供水管网37公里、燃气管网51公里，建成烈山、杜集片区污水转输管网42公里。建设公共自行车三期工程，改造提升老旧公厕59座，首批新能源公交车投入使用、首条旅游公交专线开通运行。新型智慧城市在政务服务、城市管理等方面实现深度融合，无线城市建设初见成效。建成凤凰公园等18个公园和重要绿化节点，烈山、卧牛山山体治理加快推进，市一中东校区、市人民医院新院区开工建设，东部新城开发进一步提速。

美丽乡村建设由点到面全面铺开，农村环境“三大革命”一体化推进，5个镇政府驻地建成区、28个中心村和230个自然村环境整治成效明显。完成农村公路畅通工程415公里、改造农村危房1000户，农村电网改造升级工程全部竣工。石榴小镇、芳香小镇（试验）入选第一批省级特色小镇。全年完成棚改41300套，基本建成保障性安居工程27407套，货币化安置率达94.5%。我市棚改工作入选全省年度最具影响力十件大事，棚改模式成为全国社会治理典型案例。

****（五）扎实推进生态保护，人居环境显著改善。****启动国家森林城市创建工作，全年完成营造林6.2万亩，超额完成省下达任务。《淮北市绿化条例》正式实施。全面建立河长制，我市在全省水资源管理年度考核中居第3位，淮水北调市级配水工程具备全线通水条件。牢牢守住耕地红线，全面完成永久基本农田划定工作。以中央和省环保双督察整改为抓手，着力解决非法采石、露天物料堆场等群众反映强烈的环境突出问题，得到督察组充分肯定。全年秸秆禁烧实现零火点，首座秸秆电厂正式投产，秸秆综合利用率达86%。我市荣获中国绿色发展优秀城市，获批第三批国家低碳城市试点、首批国家气候适应型城市建设试点，濉溪县成为全国首批重点采煤沉陷区综合治理试点。央视等主流媒体对我市相山、黄里、榴园、南山等生态典型作了连续报道。抓生态建设就是抓绿色发展、补小康短板的理念更加深入人心。

****（六）统筹发展社会事业，民生福祉持续增进。****财政投入民生领域资金123.8亿元，占全部财政支出的84%。扎实开展“重精准、补短板、促攻坚”专项整改行动，落实产业扶贫、健康扶贫等重点举措，15个贫困村出列、8981人顺利脱贫。成立全省首个创业服务联盟，创业带动就业成效明显，新增城镇就业4.8万人。学前教育毛入园率提前完成省定目标，在皖北率先全面实现义务教育基本均衡目标。“三医”联动改革有序推进，“一格三员”卫生计生服务新模式获国家卫计委肯定，我市荣获全国创建幸福家庭活动示范市。成功举办第十届运动会暨第四届职工运动会、第三届残疾人运动会，全民健身与全民健康深度融合。城乡低保标准实现“七连增”，企业退休人员基本养老金实现“十三连涨”，机关事业单位工作人员养老保险制度改革全省领先。举办市民文化节系列活动，公共文化服务能力进一步提升。以喜迎十九大为契机，精心做好维稳安保工作，积极化解信访积案，群众安全感指数实现“五连升”。相山区荣获全国平安建设先进区。杜集区荣获全国首批农村幸福社区建设示范区。安全生产“铸安”行动不断深化，省属煤炭企业安全生产移交属地管理工作平稳过渡。省级双拥模范城顺利通过考核验收，食品药品安全城市创建工作积极推进。地方志、防震减灾、应急管理、保密工作获全国、全省先进，工会、共青团、妇联等作用进一步发挥，外事侨务、民族宗教、档案、机关事务、供销、科协、气象、工商联等工作持续加强。

各位代表，创建全国文明城市是全市人民的共同梦想。2017年，我们坚持全城总动员、全民齐参与，矢志不渝、接力冲刺，全面决战文明创建，我市以全国第三、全省第一的骄人成绩，成功入选第五届全国文明城市，兑现了历届市委、市政府的庄严承诺，实现了220万淮北人民的美好愿望，展现了新时代精神文明高地的淮北风采！

****（七）坚持从严从实要求，自身建设全面加强。****扎实推进“两学一做”学习教育常态化制度化，开展“讲政治、重规矩、勇担责、作表率、促崛起”专题警示教育，增强“四个意识”，落实全面从严治党各项要求。出台法治政府建设实施方案，严格执行重大事项决策规定程序和政府法律顾问制度，依法决策、依法行政水平进一步提高。自觉接受人大监督和政协监督，全年办理市人大代表议案建议78件，市政协委员提案135件。播出政风行风热线346期，推动解决群众反映问题1537个。市长热线、网民留言办复率均100%，获评全国网民留言办理工作先进单位。以“互联网+政务服务”为载体，建成市级一体并与省贯通的电子政务服务平台，2752项清单事项上线运行，网上办项率达99.4%，政务服务效率进一步提高。推进公共资源全程电子化交易，建成市县一体化公共资源交易平台。加强党风廉政建设，强化行政监察，推动审计监督全覆盖，坚决查处侵害群众利益的不正之风和腐败问题。

各位代表，2017年各项目标任务的顺利完成，标志着本届政府的正式收官。回首过去五年，面对复杂严峻考验，我们紧紧依靠全市人民，一任接着一任干、一锤接着一锤敲，开创了淮北转型发展的新局面，我市经济社会发展进入了提质提效、转型崛起的新阶段。五年的奋斗历程和生动实践也给予我们深刻启示。

****实现转型崛起，必须坚持理念为先。****把践行新发展理念与淮北实际紧密结合起来，尊重经济社会和城市发展规律，在实践中守正出新、探索新路。五年来，我们始终把淮北的发展放在全省大局来考虑，置于全国同类资源型城市的整体中把握，坚定转型发展方向不动摇。市第八次党代会创造性提出中国碳谷·绿金淮北战略，在实践中不断丰富完善，形成“一二三四五”总体发展思路，为我们找到了新时期破解转型难题的“金钥匙”，全市共识广泛凝聚，极大增强了我们加快赶超的信心和决心。

****实现转型崛起，必须坚持发展为上。****继承和发扬矢志不渝的创城精神，咬定青山不放松、不达目标不放手，逆境中坚定信心、加压奋进，顺境时乘势而上、加快发展。全市生产总值、固定资产投资、规模以上工业增加值、城乡居民人均可支配收入年均分别增长7.1%、12.9%、7.1%、7.5%、9.8%。工业化率、城镇化率分别居全省第2位、第4位。五年来，累计引进亿元以上规模项目650个，陶铝新材料、金龙电子、卓泰化工、平山电厂二期、华翊文创科教城等一批技术“世界领先”的企业和项目落户淮北，成为转型崛起的重要战略支点。

****实现转型崛起，必须坚持民生为本。****牢固树立以人民为中心的发展思想，紧紧依靠群众，广泛发动群众，从群众最盼望的事情做起，从最不满意的事情改起。五年来，全市累计投入民生领域财政资金达538.4亿元，民生工程连续九年居全省第一方阵。近两年共实施棚改项目191个、完成棚改10.5万套，群众由“蜗居”变安居。教育均衡发展和现代化加快推进，全民健身活动广泛开展，健康淮北战略全面实施，柳孜运河遗址成为我市一张世界级文化名片。城乡基本养老、基本医疗保险和居民大病保险实现全覆盖，新农合领跑全省，18.6万名国企职工医疗保险纳入市级统筹。39780人脱贫、19个贫困村出列，脱贫攻坚成效明显。我市连续八年被评为全省综治工作（平安建设）先进市，安全生产工作连续四年获省政府表彰，五年吸纳市外人口4万余人，群众安全感、幸福感指数皖北第一、全省领先的局面进一步巩固。

****实现转型崛起，必须坚持改革为要。****把改革贯穿于经济社会发展各个领域和各个环节，向改革要质量、要效率、要动力。五年来，累计承担国家级改革试点任务7项、荣获国字号荣誉9项。国家中小城市综合改革、新型智慧城市建设、社会信用体系建设在全国创造经验、成为典型。市区财税体制、规划建设管理体制改革调动了区级抓发展的积极性。“放管服”改革、商事制度改革深入推进，六大便民利企服务中心建成使用，激发了社会主体活力，累计新登记私营企业18587户、个体户66474户。市建投集团顺利重组，总资产突破800亿元，是成立之初的2.4倍，带动社会投资近500亿元，经营城市、服务发展的能力显著增强。

****实现转型崛起，必须坚持担当为荣。****大力弘扬奔着问题去、迎着困难上的工作作风，善抓大事，敢碰难事，多办实事，以担当和落实引领政府工作新常态，用苦干实干巧干实现梦想、赢得未来。五年来，我们保持战略定力，克服下行压力，沉着应对煤价巨幅波动带来的严峻考验，坚决完成煤炭去产能任务，顽强挺过经济发展的最困难时期，全面打赢稳增长保卫战和转型升级攻坚战，我市发展迎来柳暗花明又一村的光明前景，成为全国资源型城市中的一道独特风景。

各位代表，今天淮北发展所取得的全方位和开创性成就，离不开过去数十年特别是近五年来的接力奋斗、积累创造。这是省委、省政府和市委正确领导的结果，是市人大、市政协和社会各界监督支持的结果，是全市上下深入践行新发展理念，和衷共济、顽强拼搏的结果。在此，我代表市人民政府，向全市人民，向各民主党派、工商联和无党派人士、各人民团体，向中央和省驻淮单位以及人民解放军、武警官兵和政法干警，向所有关心支持淮北改革发展事业的各级领导、各位离退休老同志和广大企业家，致以崇高的敬意和衷心的感谢！

在肯定成绩的同时，我们也清醒地看到，全市经济社会发展仍存在许多突出问题和短板：经济总量不大，创新驱动能力不强，转型升级任重道远；实体经济困难较多，民营经济不发达，县区、园区发展不充分，“四化”推进不协调；城乡基础设施欠账较多，生态环境治理和保护任务繁重，民生领域还有不少短板，发展不平衡问题依然突出；政府廉政建设、作风建设、效能建设还存在一些薄弱环节。我们一定聚焦问题，精准发力，切实解决。

****二、决胜全面建成小康社会、谱写淮北现代化建设新篇章****

今后五年，是我市深入贯彻党的十九大精神，全面建成小康社会的决胜期，也是聚力实施中国碳谷·绿金淮北战略，加快转型崛起的奋进期。党的十九大开启了中国特色社会主义的新时代，作出分两步走全面建成社会主义现代化强国的战略部署，提出要发挥优势推动中部地区崛起，支持资源型地区经济转型发展，为我们带来重大历史机遇。省委、省政府高度重视皖北崛起，“34155”的新要求为淮北发展指明了方向，注入强劲动力。经过多年发展，我市综合实力持续提升，“三基三千亿”产业前景可期，城市加快由“依山建城”向“拥湖发展”跨越，现代交通、对外开放、生态环境等条件大幅改善，全国文明城市、国家中小城市综合改革试点等多重优势叠加。特别是市委确立了中国碳谷·绿金淮北战略和“一二三四五”总体发展思路，根据中央和省委决策部署，提出从现在到本世纪中叶，在决胜全面小康的基础上，实现“三个同步”，进一步激发了全市人民的热情和干劲。淮北发展已经进入新的历史方位，我们完全有信心、有能力、有条件，紧跟时代步伐，决胜全面建成小康社会，谱写淮北现代化建设新篇章。

****今后五年工作的总体思路是：****深入学习贯彻党的十九大精神，以习近平新时代中国特色社会主义思想为指导，按照市第八次党代会和市委八届五次全会的决策部署，坚定实施中国碳谷·绿金淮北战略和“一二三四五”总体发展思路，坚持稳中求进、好中求快工作总基调，坚持高质量和快速发展有机统一，坚持以供给侧结构性改革为主线，坚持以五大发展行动计划实施方案为总抓手，坚决打好防范化解重大风险、精准脱贫、污染防治三大攻坚战，在践行新发展理念中闯出新路径，在实现高质量发展上取得新突破。

****今后五年发展的总体目标是：****经济发展速度皖北领先，主要指标增幅超过全省平均水平，经济总量、均量、质量迈上新台阶，与全国、全省同步全面建成人民认可、经得起历史检验的小康社会，实现转型崛起。

****——提升创新驱动力，建设转型崛起新淮北。****科技创新成为经济增长的主导力量，基本形成实体经济、科技创新、现代金融、人力资源协同发展的创新型现代化产业体系，建成创新型城市。到2022年，非煤产业占工业比重超过80%，高新技术产业增加值占规模以上工业比重达40%，现代服务业占比显著提升，农产品加工产值与农业总产值之比达6:1。铝基、碳基和硅基新材料产业集群发展，总产值均突破千亿元，成为国内知名的新材料产业基地。

****——增强发展协调性，建设全域统筹新淮北。****城市综合承载力显著提升，乡村振兴战略全面实施，县区经济提速发展，城乡资源配置更加均衡，园区改革创新步伐加快，基本形成市域一体、县区联动、产城融合的协同发展新格局，建成区域中心城市。到2022年，中心城区建成区面积超过110平方公里，户籍人口城镇化率达65%，80%以上中心村达到美丽乡村建设标准。濉溪县综合实力进入全省前五、三区实现争先进位，对全市贡献更加突出。国家级开发区、国家级高新区实现重大突破，成功创建全国资源枯竭可持续发展示范市。

****——提高绿色竞争力，建设生态样板新淮北。****环境综合治理深入推进，资源能源开发利用率大幅提高，主要污染物达标排放，空气质量持续改善，黑臭水体全部消除，城市“双修”和海绵城市建设取得实效，绿色经济初具规模，山水生态园林城市特色凸显。到2022年，森林覆盖率达到30%以上，空气质量优良天数保持在80%以上，单位生产总值能耗下降16%，成功创建国家森林城市、国家环保模范城市，成为全国资源型城市绿色发展样板。

****——打造开放新高地，建设包容共赢新淮北。****全社会大开放意识牢固树立，深度对接国家“三大战略”，全方位融入中原经济区、淮河生态经济带。综合交通体系更加完善，大通道、大平台、大通关体系基本建立，产业、企业、城市、人才国际化进程全面提速。区域经贸合作持续深化，外贸进出口量增质优，开放型经济规模大幅提高。到2022年，成为苏鲁豫皖四省交汇区域开放创新新高地。

****——增强群众幸福感，建设富民惠民新淮北。****城镇居民收入与经济增长同步，农村居民收入快于经济增长。贫困人口全部脱贫、贫困村全部出列。劳动就业更加稳定充分、现代教育体系基本建成、全民科学素质建设积极推进、群众精神文化生活更加充盈、公共卫生服务更加健全、社会保障体系全面覆盖。法治淮北、平安淮北、信用淮北建设取得明显成效，群众安全感和幸福指数持续攀升，成为全国社会治安综合治理优秀城市。文明城市共同缔造理念深入人心，城乡发展全面实现共建共享。

****三、砥砺前行、转型崛起的新征程****

2018年是全面贯彻落实党的十九大精神的开局之年，也是新一届政府任期的第一年。全市经济社会发展的主要预期目标是：生产总值增长7.5%以上，财政收入增长8.5%，固定资产投资增长9%，社会消费品零售总额增长10.5%，外贸进出口总额增长8%，城乡居民人均可支配收入分别增长7.5%、8%，城镇登记失业率控制在4.2%以内，节能减排完成省定目标。重点做好八个方面的工作：

****（一）突出制造强市战略，着力构建现代产业体系****

牢固树立工业立市、制造强市理念，突出创新引领发展第一动力，推动质量变革、效率变革、动力变革，加快转变发展方式，促进产业转型升级。

深化供给侧结构性改革，促进实体经济健康发展。完成煤炭去产能90万吨，统筹做好职工分流安置、“三供一业”分离移交、退出矿井资源盘活等工作。落实支持城镇居民住房消费、进城农民和务工人员购房奖励补贴相关政策，发展住房租赁市场，促进房地产市场平稳健康发展。推进企业市场化、法治化债转股，引导企业进入多层次资本市场，积极开展直接融资。全面落实实体经济企业降成本各项政策，持续降低企业制度性交易成本和用能、物流成本。常态化开展“四送一服”“三个一百”帮联企业专项行动，打通服务企业“最后一公里”。调整政府性投资项目导向，向实体经济倾斜，加大补短板项目建设。全年组织实施重点项目605个、完成投资482.7亿元。

加快“三重一创”建设，促进新兴产业加速发展。坚持把“三基三千亿”工程作为引领产业转型升级的一号工程，突出抓好100万吨碳基新材料、陶铝新材料、金龙机电、清华紫光等项目建设，抢占产业制高点。战略性新兴产业产值增长18%。深入推进平山电厂二期、口子产业园、完美生物、华孚产业园、锂电池产业园等工业项目建设，打造发展新引擎。全年新增规模以上工业企业80家，规模以上工业增加值增长8%。深化与中科院、上海交通大学等合作，构建以企业为主体、市场为导向、产学研深度融合的产业创新平台和转化体系。全年新认定高新技术企业25家，高新技术产业增加值增长10%以上。积极争创国家知识产权示范城市和省创新型城市。发挥市产业扶持基金、重大新兴产业投资基金的引导作用，建立覆盖企业全生命周期的投资基金体系，畅通实体经济融资渠道。建立高层次人才储备金制度，全年引进高层次科技创新人才团队2-3个，高层次创新人才100名以上。实施新一轮技术改造专项行动，完成产业升级和技改项目140个；实施“互联网+制造”行动，新增省智能工厂、数字化车间5个；实施增品种、提品质、创品牌“三品”行动，推进企业质量、品牌、标准升级；实施工业节能与绿色发展专项行动，培育创建绿色工厂、绿色产品、绿色园区；实施服务型制造专项行动，培育省级示范企业2家。加快东华中小企业云服务智慧产业园建设。争创国家级双创空间1个。整合政府数据资源，培育发展大数据产业，建设大数据大厦，入驻企业10家以上。

打造区域休闲旅游目的地，促进现代服务业繁荣发展。依托中湖等城区湖带治理，谋划建设“湖文化”国家级旅游休闲度假区。设立市级旅游业发展专项资金，组建旅游文化传媒集团，形成多元投入机制。发挥口子文博园、华翊文创科教城、隋唐运河古镇、四季榴园等示范效应，推动旅游与教育、科技、文化等产业深度融合。积极创建国家全域旅游示范区，办好石榴文化旅游节、段园葡萄采摘节等节庆活动，加快将旅游业培育成为支柱产业。推动传统商圈提档升级，建成万达广场、中农批淮北大市场等大型商业区、专业市场。发展商贸流通业，支持跨境电商平台发展，完善县乡物流配送网络，推动青龙山等商贸物流园区标准化建设。打造创新型公共服务平台，争创省级服务业集聚区1个，培育规模以上现代服务业企业16家。

****（二）统筹城乡协调发展，着力提升城市品质****

以城乡协调为主攻方向，创新城市发展方式，加大基础设施建设投入，纵深推进文明创建，提升协调发展整体水平。

****注重协同联动，完善现代交通体系。****加强综合交通体系规划建设，积极推进徐淮阜高速公路项目，淮宿蚌城际铁路、通用机场实质性开工，提高快速通达能力，进一步拓展发展空间。开工建设G344东灵线、G237淮六路、淮徐快速通道、京台高速出入口连接线东移、S337西临路工程，加大S101合相路二期、S254古毛路二期推进力度。加快韩村港建设，实现浍河全线通航。

****加强城市建设，提升综合承载能力。****编制完成《淮北市城市总体规划（2016-2035年）》，优化城市空间布局，指导城市未来发展。坚持全球视野、国际标准，编制城区中心湖带概念性规划和城市设计，彰显城市特色风貌，加快拥湖发展步伐。完善城乡骨干路网，建成东部新城8条道路、高铁新区4条道路及古城东路、龙山北路，实施泉山隧道工程，打通桓谭路、渠沟东路等断头路，开工建设环朔西湖路、龙昌路、青年路、杨河路等滨湖道路。建成地下综合管廊15.4公里、海绵城市10平方公里。实施新一轮棚改三年计划，全年棚改35205套。加大老城区改造力度，完成南黎花园等8个老旧小区改造。全面启动老城区雨污分流工程，彻底解决历史遗留问题。加快市一中东校区、市人民医院新院区、市规划展示馆等重点项目建设，提升东部新城建设品质。加紧实施南部次中心起步区棚改和公共服务项目，加快高铁新区、凤凰新城基础设施建设，谋划建设双堆集中心集镇新城。加强智慧城市建设，构建智慧淮北大数据与云平台，促进大数据在城市管理、民生服务等领域广泛应用。推进无线城市建设，实现市区主要公共区域WIFI全覆盖。

****拓展创建内涵，共同缔造全域文明。****以社区为中心，构建“十分钟便民生活圈”，新建改造一批标准化菜市场、公厕、街心公园等生活服务配套设施，下大力气解决群众买菜难、出行难、如厕难、停车难、休闲难，提升便民服务水平。坚持以市民为主体的创建理念，发动全员参与，引导市民自觉遵守“淮北文明18条”，持续开展交通秩序、违规养犬、露天烧烤等专项整治，让文明渗入城市“肌理”，融入百姓生活。完善文明创建长效机制，推动文明创建由城市向乡村拓展，实施“两治三改”三年专项行动，深化“三线三边”治理，坚持不懈推进“厕所革命”，补齐影响群众生活品质的短板。深入推进农村环境“三大革命”，改造垃圾中转站23座，实现垃圾清扫保洁全覆盖，改建旱厕9万座，显著改善农村人居环境。

****实施乡村振兴战略，促进农业农村优先发展。****健全城乡融合发展体制机制，吸引资本、技术、人才等要素向乡村流动。加大“三农”投入，设立财政扶持资金，引导土地适度规模流转，建设一批优质农产品基地、培育一批现代农业产业化联合体，发展一批农业新业态，促进一二三产融合发展。农产品加工产值达670亿元。支持濉溪县创建国家现代生态农业产业化示范县。支持和鼓励农民就业创业，千方百计拓宽增收渠道。推进美丽乡村建设，集中抓好“五项建设”的巩固提升，改善农村基础设施。铺设供水管网42公里，改造危房787户，解决7万人的饮水安全问题。加强农村“四好”公路建设，新建农村畅通工程142公里。注重保护村容村貌，挖掘乡风民俗，促进乡风文明。因地制宜打造石榴小镇、芳香小镇、葡萄小镇等一批特色小镇，构筑产业发展载体和创新创业空间。

****推进城乡一体，加快振兴县区经济。****落实和完善支持政策，鼓励县区摆脱传统路径依赖，突出特色、壮大产业，发展新经济，培育新动能。牢固树立工业强县（区）、产业兴县（区）理念，支持濉溪县以铝基高端金属材料产业基地和南部次中心建设为重点，发展铝基新材料、装备制造业和农副产品深加工产业集群；相山区发挥主城区优势，打造现代服务业和绿色食品产业强区；杜集区抢抓高铁机遇，发展现代商贸物流和高端装备制造业；烈山区依托东部新城建设，打造现代物流产业和生态休闲旅游强区，形成多点支撑、多极带动、竞相发展格局。

****（三）深化环境综合整治，着力打造生态样板****

牢固树立“绿水青山就是金山银山”的发展理念，加强生态治理和环境保护，解决环境突出问题，实现人与自然和谐发展。

****提升生态文明建设水平。****围绕国家森林城市创建目标，以提高森林覆盖率为重点，实施大规模绿化行动，申报相山国家级森林公园。促进林业增绿增效，完成营造林4.6万亩。实施主干道绿化修复提升工程，完成创新大道、梧桐中路、沱河中路高标准绿化，打造龙山路万亩花海。加强生态保护修复，完成烈山、卧牛山山体修复工程，加快建设中湖国家级湿地公园，开工建设花山公园、泉山原生态公园、朔西湖郊野公园，为全市人民提供更多的优质生态产品。

****推进重点领域污染防治。****强化问题导向，压实主体责任，确保中央和省环保督察反馈意见整改到位、问题“清仓见底”。持续抓好大气污染防治，采取管控施工扬尘、禁放烟花爆竹、淘汰燃煤锅炉和黄标车等综合措施，切实降低PM10浓度，打赢蓝天保卫战。深入实施“水十条”，加强河流出界断面水质监管，组织实施不达标水体达标行动，确保饮用水源水质达标率100%。强力推进黑臭水体整治，完成相阳沟、跃进河等7条河道治理。全面实施“土十条”，加强土壤污染防治和废弃矿山环境整治，积极申报国家“山水田林湖草”综合治理项目。

****健全生态文明制度。****稳步推进国家低碳城市试点、气候适应型城市试点建设，总结经验，创造亮点。加强重点生态区、水源地保护，严格落实河长制和最严格水资源管理制度。全面推行林长制。加强闲置、低效用地清理处置，促进土地节约集约利用。强化工业、建筑、交通、公共机构等重点领域节能管理，支持新能源汽车、绿色建材推广应用，降低能源消耗强度。推进餐厨垃圾无害化处理，扩大生活垃圾分类试点范围，生活垃圾填埋场一期工程年内正式投入运行。

****（四）坚持招商带动战略，着力打造开放高地****

围绕打造苏鲁豫皖四省交汇区域开放创新新高地目标，加大招商引资力度，促进园区转型升级，形成全面开放新优势。

****强力推进招商引资。****强化政府主导招商，鼓励市场主体招商，加大专业招商力度。瞄准京津冀、长三角、珠三角等重点区域，积极承接产业转移，全年引进亿元以上规模项目170个以上。广泛学习宣传王浩伟同志先进事迹，促进招商引资与招才引智有机结合。采取集中新建、商品房配建、企业自建等形式，规划建设一批人才公寓，更好地引进人才、服务人才。完善招商引资政策，对重大招商项目实行“一企一策”“一事一议”，鼓励优质企业引进战略投资者、高端智库。建立政区合作发展利益分享机制，加大对区级招商项目的财税扶持力度，提高招商实效。强化全员服务理念，大力发扬“店小二”精神，让在淮企业兴业愉快、发展愉快、生活愉快。

****大力促进园区转型升级。****以产城一体、宜业宜居、特色发展为导向，加大对园区基础设施建设和公共服务平台等投入，推进相关体制机制创新，优化园区功能布局，推动工业园区向现代城区转型。完善园区公共服务配套，新建市科创中心，促进产品研发、企业孵化和推广应用。新建标准化厂房50万平方米，满足企业入驻需求。盘活闲置厂房、仓库等，兴办众创空间，建设公共创新平台。实施龙湖高新区污水处理厂扩建工程、新型煤化工基地污水处理厂工程，建设基地南路，补齐基础设施短板。积极推进碳谷科技新城产业规划编制工作，加快产业导入，打造产城融合示范区。支持濉溪经济开发区争创国家级开发区，市经济开发区争创国家级高新区。

****积极扩大对外合作。****坚持引进来和走出去并重，深度对接国家“三大战略”，主动参与淮河生态经济带建设，加强与省际省内城市联动互动。加快对外平台建设，建成金龙电子自用型海关监管仓库。围绕青龙山现代物流基地、传化物流园、公用型保税仓库，建设全市贸易便利加工出口区。积极申报设立淮北海关，推进贸易便利化，打造对外开放新平台。

****（五）纵深推进全面改革，着力释放发展活力****

加大重点领域和关键环节改革力度，以小切口推动大变革，进一步激发市场主体活力和社会创造力。

****创优“四最”营商环境。****以“互联网+政务服务”为载体，对政务服务大厅进行智能化改造，加强市级一体化平台建设，融入全省“一张网”，推进业务流程再造。实施“13550”行动，推进不见面审批，年内实现市场主体和群众到窗口办事“最多跑一次、多次是例外”。动态调整政府权责清单，打造权责清单“升级版”。探索开展行政执法人员二维码监督，推进监管标准化。深化商事制度改革，推进“多证合一” “证照分离”“先照后证”，降低市场准入制度性成本，完善市场监管体制，促进各类市场主体繁荣发展。

****深化财政金融体制改革。****科学划分财政事权和支出责任，建立规范透明的预算制度，强化财政资金绩效管理。加快市场化融资步伐，拓宽社会合作领域及融资渠道，继续实施一批PPP项目。推进国有企业混合所有制改革，完善国资监管体系，确保国有资产保值增值。严格控制政府债务规模，规范政府举债方式，防范化解财政金融风险。深化金融改革发展，持续推广税融通、新型政银担合作、续贷过桥专项资金等业务，增强金融服务实体经济能力。深化招投标体制改革，推行公共资源交易平台服务标准化。深化市建投集团改革，壮大优质资产规模，市金控集团经营性收入突破100亿元。

****深化农村综合改革。****深入推进“三权分置”和“三变”改革试点，巩固农村承包地确权登记颁证成果。大力推进农村集体产权制度改革和农业水价改革，全面完成农村集体资产清产核资工作。积极开展“一户一田”改革试点，保障农民财产权益，壮大集体经济。加快市县农村产权交易市场建设。

****大力发展民营经济。****始终坚持“两个毫不动摇”，全面落实支持民营经济发展各项政策，优化法治环境、金融环境、创新环境，构建“亲”“清”新型政商关系，激发和保护企业家精神。大力弘扬劳模精神、工匠精神，推动大众创业、万众创新，为更多的创业者、劳动者搭建人生出彩的平台。实施市场主体增量工程，新增私营企业5000户、个体工商户1.2万户。推动“小升规、上台阶”，培育龙头企业36家，新增“专精特新”企业15户，民营经济增加值占生产总值比重提高1个百分点。

****（六）扩大优质公共服务供给，着力实现共建共享****

扎实推进以需求为导向的民生工作，补短板、强弱项、兜底线，不断增强群众的获得感和幸福感。

****打赢脱贫攻坚战役。****深入实施脱贫攻坚“十大工程”，推广“四带一自”产业扶贫模式，加大教育扶贫力度，优化健康脱贫“351”“180”政策，做到精准扶贫全覆盖。加大薄弱环节补短板力度，推进基本公共服务均等化。完善防范返贫机制，落实后续帮扶措施，确保稳定脱贫。动员全社会力量参与，深化结对帮扶、企业帮扶，确保3个贫困村出列、3983人如期脱贫。

****加大民生保障力度。****坚持量力而行，尽力而为，加强省、市重点民生工程建设，完善政府主导、多方参与的民生投入机制，切实提高民生工程质量和效益。坚持以创业带动就业，深入实施“四进四扶”计划，新增城镇就业4万人。构建和谐劳动关系，保证农民工工资按时足额支付。扎实推进养老保险全国统筹，建立城乡统一的基本医疗保险制度和大病保险制度。

****统筹发展社会事业。****实施第三期学前教育行动计划，推动义务教育由基本均衡向优质均衡发展。提高普通高中办学质量，加快高中阶段教育普及步伐。推进教育信息化建设，扩大优质教育资源覆盖面。创新发展现代职业教育，深化产教融合、校企合作，建设技工大市。积极参与大运河文化带建设，注重文化遗产保护利用传承，打造运河文化品牌。加强公共文化服务体系建设，促进文化惠民。推进体育强市、健康淮北建设，积极创建全省卫生城市。深化医药卫生体制改革，构建家庭医生、医共体和医联体协同服务体系，促进医疗卫生工作重心下移、资源下沉，更好满足群众健康需求。加强妇幼保健计划生育服务能力建设，提高妇女儿童健康水平。巩固全国创建幸福家庭活动示范市成果。做好第四次全国经济普查工作。构建现代养老服务体系，推进医养结合，高品质规划建设朔西湖健康小镇，打造全国一流的健康养老示范基地。

****（七）不断创新社会治理，着力增进社会和谐****

以法治淮北、平安淮北、信用淮北建设为主线，完善社会治理体系，促进社会大局和谐稳定，争创全国社会治安综合治理优秀城市。

****守护公共安全底线。****大力推进“智慧公安”建设，开展“守护平安”系列行动，实施“雪亮工程”，不断完善信息化、立体化社会治安防控体系，坚决打击各类违法犯罪活动。创建省级食品药品安全示范城市，保障群众饮食用药安全。加强突发事件应急管理，开展群众性应急救护知识宣传培训，提高全社会应急意识和避险能力。

****筑牢安全生产基础。****深入推进安全生产领域改革发展，强化企业主体责任，完善监管监察体制，构建安全风险“六项机制”和隐患排查治理双重预防机制。开展安全生产“铸安”行动和本质安全提升年活动，抓好煤矿、危化品、道路交通等重点行业和领域安全，确保安全生产形势稳定。

****创新社会治理方式。****深入实施“七五”普法规划，弘扬法治文化，优化公共法律服务。完善预防和化解社会矛盾机制，深化阳光信访、责任信访、法治信访建设，解决事关群众切身利益的实际问题。以社区（自然村）为基本单元，建立居民（村民）理事会，推进居民（村民）自治。积极创建全国和谐社区建设示范城。扎实推进信用体系建设示范市工作，完善守信联合激励和失信联合惩戒机制，以政务诚信带动商务诚信和社会诚信。

完善国防动员体系，加强全民国防教育，开展“双拥在基层”和“四方共建”主题双拥工作，巩固发展军政军民团结良好局面。

****（八）加强政府自身建设，着力打造人民满意政府****

新形势、新任务对政府工作提出了更高的发展定位和要求。我们将始终不忘初心，牢记宗旨，加快转变政府职能，全面提升政府治理能力、公信力和执行力。

****强化对标看齐。****坚定维护以习近平同志为核心的党中央权威和集中统一领导，严守政治纪律和政治规矩，树牢“四个意识”，增强“四个自信”。深入学习宣传贯彻党的十九大精神，切实做到学懂、弄通、做实，更加坚定自觉地用习近平新时代中国特色社会主义思想武装头脑、指导实践、推动工作。

****恪守为民宗旨。****坚持人民主体地位，把人民对美好生活的向往作为政府永恒的奋斗目标。大兴调查研究之风，深入基层一线，倾听群众呼声，找准短板弱项，从实事小事抓起，持之以恒，久久为功。推进政府服务热线资源整合，强化“12345”市长热线功能，及时回应社会关切，为群众排忧解难。

****严格依法行政。****切实履行法治政府建设职责，坚持法定职责必须为，法无授权不可为。健全依法决策机制，严格落实重大事项合法性审查和重大执法决定法制审核制度。全面推进政务公开，建成政府网站集约化云平台。自觉接受人大监督和政协监督，让在监督下开展工作成为政府全体人员的自觉和习惯。

****坚守廉政底线。****认真落实党风廉政建设“两个责任”，把纪律建设贯穿于政府工作全过程，提升全面从严治党水平。坚持用制度管权、管人、管事，加大审计监督力度，从源头预防腐败。全面落实中央八项规定精神及省市《实施细则》，驰而不息纠正“四风”，坚决反对形式主义和官僚主义。持续加强机关效能建设，严惩庸政懒政怠政，提高政府工作质量和效率。

****提升成事本领。****坚持奔着问题去、迎着困难上，磨练担当宽肩膀，历练成事真本领。建立健全正向激励和容错纠错机制，鼓励政府系统全体人员敢于担当、敢于负责、敢于督查、敢于成事。健全执行、监督、考核、奖惩机制，以踏石留印、抓铁有痕的韧劲和钉钉子精神抓落实，确保中央和省市各项决策部署全面贯彻、落地生根。

三山六湖风景秀，文明相城家园美。220万淮北人民的信任和期盼，是激励我们勇往直前、永不懈怠的最大动力。让我们在中共淮北市委的坚强领导下，真抓实干，奋发进取，为我市全面建成小康社会，建设中国碳谷•绿金淮北，打造全国资源型城市转型样板而努力奋斗！
